# Lowering salivary pH with sugar‐containing gum augments salivary nitrite production and blood pressure reduction with dietary nitrate (beetroot juice)

**DOI:** 10.1002/bcp.70640

**Published:** 2026-06-18

**Authors:** Andrew J. Webb, Harriet Clift, Callum Hill, Nur Mousa, Navanithan Arun Jayaraj, Jasmine Quraishi, Charlotte E. Mills, Kevin O'Gallagher

**Affiliations:** ^1^ King's College London British Heart Foundation Centre, School of Cardiovascular and Metabolic Medicine and Sciences, Department of Clinical Pharmacology London UK; ^2^ Clinical Research Facility Guy's and St Thomas' NHS Foundation Trust London UK; ^3^ Department of Nutritional Sciences, School of Life Course Sciences King's College London London UK; ^4^ Food and Nutritional Sciences University of Reading Reading UK

**Keywords:** blood pressure, nitrate–nitrite–NO pathway, salivary pH

## Abstract

**Aims:**

The nitrate–nitrite–nitric oxide (NO) pathway has a wide range of effects including blood pressure (BP) regulation. Nitrate, produced endogenously, and absorbed from dietary sources (e.g., beetroot), is concentrated in/secreted by the salivary glands and reduced by lingual bacterial nitrate reductases to nitrite (the ‘enterosalivary circulation’). Following systemic absorption, nitrite is further reduced to vasodilating NO intravascularly. The role of salivary pH in nitrate reduction has mainly been studied in isolated oral installation studies, rather than systemic studies employing the enterosalivary circulation. Our aim was to determine the acute relationship between salivary pH, salivary nitrite production, plasma [nitrite] and the BP‐lowering effect of dietary nitrate, by altering salivary pH using sugar‐containing chewing gum (*vs*. sugar‐free chewing gum).

**Methods:**

We performed a 7‐h crossover study, with 14 healthy volunteers consuming beetroot juice (Beet‐It®, 70‐mL, ~400‐mg nitrate) and randomized to initially chew either sugar‐containing acidic chewing (bubble) gum or sugar‐free non‐acidic chewing gum, with the alternate gum on the second visit.

**Results:**

Sugar‐containing gum decreased salivary pH by 1.4 ± 0.09 *vs*. sugar‐free gum (*p* < .0001). While there was no significant effect on salivary nitrate secretion or plasma [nitrate], salivary nitrite production was increased by 45% (30.0 ± 11.0 μmol·h^−1^; [*p* < .01]), with plasma [nitrite] increasing by 25% (222.2 ± 50.3 nmol·L^−1^; [*p* = .0001]). Systolic and diastolic BP were decreased by 2.7 ± 0.5 and 1.9 ± 0.5 mmHg, respectively (both *p* < .0001).

**Conclusion:**

Acutely lowering salivary pH (with sugar‐containing gum) augments salivary nitrite production, plasma [nitrite] and BP‐lowering with dietary nitrate. This mechanism has potential to enhance other nitrate–nitrite–NO pathway effects, for example, exercise performance.

## Graphical abstract

Acutely lowering salivary pH (with sugar‐containing acidic gum *vs*. sugar‐free gum) augments salivary nitrate reduction to nitrite, plasma nitrite concentration, and blood pressure lowering with dietary nitrate. Modifying salivary pH reveals a mechanism by which the nitrate‐nitrite‐NO pathway may be upregulated/inhibited, with potential for other related effects, for example, exercise performance.

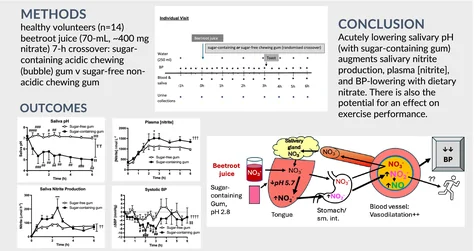

**What is already known about this subject**

- Nitrate (endogenously and dietary derived, e.g., beetroot juice) lowers blood pressure via the nitrate–nitrite–nitric oxide pathway.
- Reduction of salivary nitrate to nitrite by lingual bacterial nitrate reductases to nitrite is a critical bioactivation step.
- The acute pH‐dependency of nitrate reduction awaits full characterization in systemic (enterosalivary) studies.

**What this study adds**

- Acutely lowering salivary pH (with sugar‐containing gum) enhances salivary nitrate reduction to nitrite, circulating (nitrite) and blood pressure lowering with dietary nitrate.
- Modifying salivary pH reveals a potential mechanism by which the nitrate–nitrite–NO pathway may be upregulated/inhibited.
- This relationship differs between our systemic (enterosalivary) studies *vs*. isolated oral installation studies.

## 1 INTRODUCTION

The nitrate–nitrite–nitric oxide (NO) pathway has been demonstrated to have a wide range of effects particularly in the cardiovascular system. This includes blood pressure (BP) regulation and maintenance of endothelial/vascular and platelet function,^1^, ^2^ prevention of atherosclerosis,^3^, ^4^ and renal dysfunction,^5^ enhancement of exercise performance in healthy volunteers/athletes,^6^ and in patients with co‐morbidities such as chronic obstructive pulmonary disease.^7^, ^8^ Inorganic nitrate (NO_3_^−^) derived from the oxidation of endogenously‐produced NO from NO synthases (NOS) and dietary sources (e.g., green leafy vegetables and beetroot) is concentrated in, and continuously secreted by, the saliva glands into the oral cavity. There, nitrate is reduced to nitrite (NO_2_^−^) by lingual commensal bacteria possessing nitrate reductases (Figure 1). Upon swallowing saliva, nitrite is absorbed systemically and further reduced to vasodilating NO in the circulation. Interrupting this enterosalivary circulation by avoiding swallowing and instead spitting out the (nitrite‐containing) saliva, blocks the increase in plasma [nitrite] and the decrease in BP otherwise observed following a nitrate load.^9^ Similarly, rinsing the mouth with anti‐bacterial mouthwash before a dietary nitrate load abolishes nitrate reduction to nitrite, also blunting the rise in plasma [nitrite].^10^ Mouthwash also increases BP *vs*. control, in a manner inversely correlated with the diminished plasma [nitrite].^11^

**FIGURE 1:**
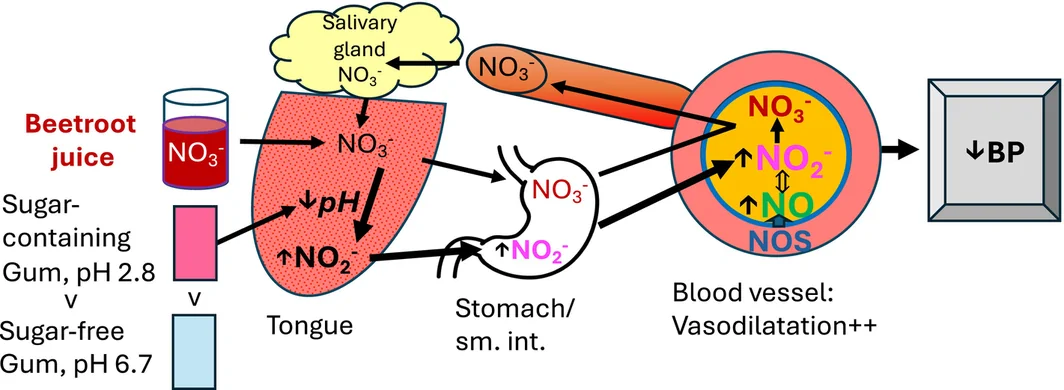
The nitrate (NO_3_^−^)–nitrite (NO_2_^−^)–nitric oxide (NO) pathway and the proposed acute influence of sugar‐containing acidic *vs.* sugar‐free gum on lowering salivary pH and enhancing nitrate reduction to nitrite by bacterial nitrate reductases on the tongue. Nitrite is absorbed via the upper small intestine into the circulation increasing plasma [nitrite], which is reduced to NO resulting in more vasodilatation and blood pressure (BP) lowering. Nitrate is derived from dietary sources (e.g., beetroot juice) and from the oxidation of nitric oxide synthase (NOS)‐derived NO.

We have previously demonstrated that, in an acute study, the combination of grapefruit juice and (active, nitrate‐containing) beetroot juice significantly increases salivary pH compared to the combination of water (as control) with active beetroot juice.^12^ This increase in salivary pH was associated with a decrease in salivary nitrite production, and in the ensuing plasma [nitrite]. Salivary pH is also increased (from pH 7.0 to 7.5) with chronic ingestion (15 days by 46 volunteers) of nitrate as beetroot juice. However, this was associated with increases in both salivary [nitrate] and [nitrite].^13^ Chronic nitrate ingestion also induces changes in the oral microbiota,^14^ associated with increases in salivary pH,^15^, ^16^ likely conferring beneficial effects on periodontal/gingival health.^13^, ^17^, ^18^

In contrast to our whole‐body/systemic grapefruit juice study involving the enterosalivary circulation,^12^ other acute studies have mainly employed isolated models, with oral installations or incubations of inorganic nitrate with pH‐buffer solutions. Such studies appear to generate the opposite relationship between oral pH and nitrate reduction/nitrite formation. For example, peak nitrate reduction has been described at pH 8.0 (range tested pH 5.5–8.0),^19^ pH 7.3 (range tested: pH 6.8 to 7.3)^20^ and pH 8.1 ± 0.1 (*vs*. 7.3 ± 0.1).^21^

Thus, interventions that acutely increase salivary pH appear to be associated with decreased salivary nitrate reduction in a whole‐body/systemic study (employing the enterosalivary circulation) but with increased nitrate reduction in oral installation models. We therefore hypothesized that lowering salivary pH in a whole‐body/systemic study would enhance salivary nitrate reduction (Figure 1). It has previously been demonstrated that sugar‐containing chewing gum significantly decreases salivary pH compared to both baseline and sugar‐free chewing gum,^22^ representing a method to lower salivary pH. The Aim of this study was to further determine the relationship between salivary pH, salivary nitrite production, plasma [nitrite] and BP, following a dietary nitrate load, by decreasing salivary pH using sugar‐containing chewing gum.

## 2 METHODS

### 2.1 Approvals

Approvals for this study were obtained from the South‐East London Research Ethics Committee (REC) (10/H0802/52) and from Guy's and St Thomas' Research & Development Department. Written informed consent was obtained from all volunteers prior to commencing any protocol‐related procedures.

### 2.2 Study design

A two‐visit randomized, crossover intervention design was used. For 24 h prior to study visit days, volunteers were asked to avoid strenuous exercise, smoking, use of mouthwash and to consume similar foods for breakfast, lunch and dinner, avoiding high‐nitrate foods (e.g., green leafy vegetables [spinach, lettuce, rocket and beetroot] and preserved meats [ham, salami, sausages and bacon]) and to fast for 12 h before the study. On each visit, and following an hours' equilibration period from (*t* = −1 h to *t* = 0 h), volunteers consumed a 70‐mL shot of concentrated beetroot juice containing ~0.4‐g inorganic nitrate (‘BEET IT® SPORT’ James White Ltd, Suffolk, UK), the same formulation and dose that we have used in our previous single dose,^12^ and chronic studies,^3^, ^23^ producing significant changes in nitrate and nitrite kinetic parameters, and haemodynamic parameters, diluted with 180 mL of low‐nitrate water (<0.1 mg/L, Buxton® Mineral Water, UK); 250 mL of this water was also provided at all the other hourly time points (‘optimised hydration protocol’); see Figure 2. Volunteers were given two slices of toasted wholemeal thick brown bread just after *T* = 3 h. Volunteers were asked to chew gum during each study visit. The type of gum was randomized to initially either sugar‐containing acidic (pH 2.84 ± 0.4 [SD]) chewing gum (Seriously Strawberry Hubba Bubba Bubble Gum [‘Hubba Bubba®’], Wrigley Company, Plymouth, UK), which lowers salivary pH,^22^ or sugar‐free non‐acidic (pH 6.71 ± 0.01) chewing gum (Extra Peppermint [‘Extra®’], Wrigley Company, Plymouth, UK) as control, with a neutral effect on salivary pH.^22^ The alternate gum was chewed on the 2nd visit. Volunteers were asked to chew gum throughout most of the duration of the experiment, changing the piece of gum every 20–30 min (each piece is designed to be chewed for at least 20 min according to the packaging label). Volunteers were allowed to take short breaks from chewing the gum (~5 min per hour) after they had provided saliva samples and during BP measurements (see below for details).

**FIGURE 2:**
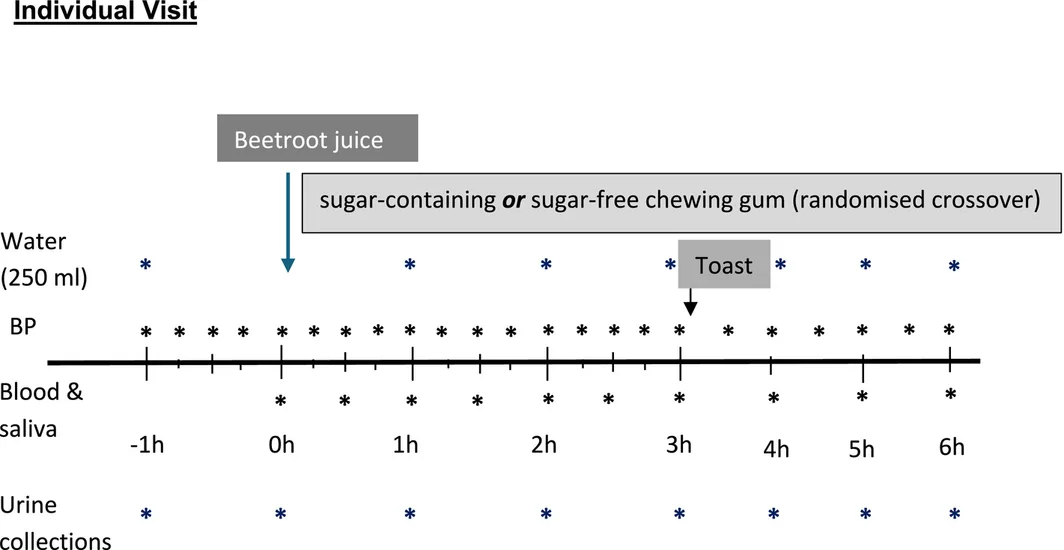
The full individual study visit protocol, showing the timing of water and beetroot juice ingestion, blood pressure (BP) measurements, blood, saliva and urine collections, indicated by the asterixis (*) against the timeline. The volunteer had been randomized to chew either sugar‐containing acidic gum or sugar‐free gum from *t* = 0–6 h, returning at an interval of at least 7 days to chew the alternative gum (crossover design). The initial eight volunteers (first cohort) completed the study to *t* = 3 h; the next six volunteers (second cohort) completed the full protocol.

BP was measured in triplicate every 15 min from 1 h (*t* = −1 h) before ingestion of the juice intervention at (*t* = 0 h) and until 3‐h post‐ingestion, and at 30‐min intervals thereafter. The BP data value for a given time point was recorded as the average of the three triplicate readings. The volunteer was seated throughout BP measurements and asked to keep their legs uncrossed and to refrain from chewing gum during BP recording. Mean arterial pressure has previously been found to increase whilst chewing gum.^24^

### 2.3 Sample collection

Blood and saliva samples were taken immediately prior to ingestion of beetroot juice, then at 30‐min intervals for 3 h, and hourly for a further 3 h. At time points where BP and blood samples were taken, BP recordings were taken prior to blood sampling.

At each blood draw, 6 mL of venous blood was drawn into a chilled syringe then transferred to a chilled green lithium heparin tube (Vacutainer®, BD). Samples were immediately centrifuged at 4°C and 2000 × *g* for 5 min (MIKRO 220R, Hettich, Germany), after which plasma was collected.

For the collection of saliva, volunteers were asked to avoid swallowing saliva for 5 min and instead to drool all saliva into a collecting tube. The volume of saliva collected was recorded.

All plasma, saliva and urine samples were divided into two chilled 2‐mL tubes and stored at −80°C until biochemical analysis.

### 2.4 Sample analysis

Plasma and saliva samples were analysed for nitrate and nitrite concentrations using chemiluminescence, as we have described previously.^25^, ^26^

### 2.5 Data analysis

Data were analysed using GraphPad Prism 10.6 (GraphPad Software Inc.). All data are expressed as mean ± SEM unless otherwise stated. Data (if normally distributed) were compared by two‐way analysis of variance (ANOVA) (paired analysis as a crossover study) and one‐way ANOVA (within‐intervention) as appropriate, with Sidak's post‐test. For non‐normally distributed data, non‐parametric statistics (Wilcoxon matched pairs signed rank test followed by one‐way ANOVA for within‐group comparison to baseline) were used. *P* < .05 was considered statistically significant.

### 2.6 Sample size and duration of study protocol

In an iterative experimental medicine approach to this exploratory study, we recruited an initial eight volunteers (first cohort). This was based on one of our other (unpublished) studies, where we found that salivary nitrite production was altered by 17.7 μmol h^−1^; SD of differences 16.0, giving an effect size of 17.7/16.0 = 1.1. To detect, using repeated measures ANOVA (between factors) an effect size of 1.1 (salivary nitrite production) in a crossover study, with *α* = .05, and power (1‐*β* err prob) = 0.8. with 2 ‘groups’ (sugar‐containing *vs*. sugar‐free gum [crossover]); seven time points (0–3 h); correlation = .5; the required sample size was eight volunteers. This gave an actual power of 0.93. Power calculations were carried out using G*Power 3.1.9.2.^27^, ^28^ Once eight volunteers had been completed, we reviewed the data to guide the overall sample size required. It was initially uncertain whether volunteers would tolerate chewing gum near‐continuously for the full 6 h allowed in the protocol. For these initial eight volunteers we therefore collected data up to *t* = 3 h, but by this time point (~2.5–3 h), we typically only start to see changes in plasma [nitrite] with dietary nitrate. Thus, while a significant difference in salivary nitrite production had been demonstrated (*p* < .0001), there were not significant differences in plasma [nitrite], (*p* = .2), systolic BP (*p* = .07) or diastolic BP (*p* = .4). As no problems were encountered with chewing gum for 3 h, we elected to collect saliva, plasma and BP data until *t* = 6 h and extend the study to 14 volunteers, hence recruiting an additional six volunteers (second cohort).

### 2.7 Nomenclature of targets and ligands

Key protein targets and ligands in this article are hyperlinked to corresponding entries in https://www.guidetopharmacology.org and are permanently archived in the Concise Guide to PHARMACOLOGY 2025/26 (Alexander et al., 2025).^29^

## 3 RESULTS

Fourteen participants completed both visits. Their Demographic and Baseline Characteristics are shown in Tables 1 and 2, respectively.

**TABLE 1:** Participant characteristics. Data are expressed as median [IQR].

*N*	14
Gender (*n*, male)	6
Age, years	21 [19.75, 22]
Weight, kg	60 [59.0, 78.7]
BMI, kg/m^2^	21.7 [19.8, 23.1]

**TABLE 2:** Baseline clinical parameters of study participants. *N* = 14. Data are expressed as mean ± SD.

	Sugar‐free gum	Sugar‐containing gum
SBP, mmHg	116 ± 11	118 ± 11
DBP, mmHg	71 ± 6	73 ± 6

### 3.1 Saliva

Salivary pH was decreased by sugar‐containing (pH 5.68, 5.29 to 7.10, median + IQR) *vs*. sugar‐free gum (pH 7.15, 6.97 to 7.23), a median difference of pH 1.47 (*p* < .01); see Figure 3A. Saliva volume (over each 5‐min collection period) was increased with sugar‐containing *vs*. sugar‐free gum (*p* < .0001), with a median difference of 6.3 mL; see Figure 3B.

**FIGURE 3:**
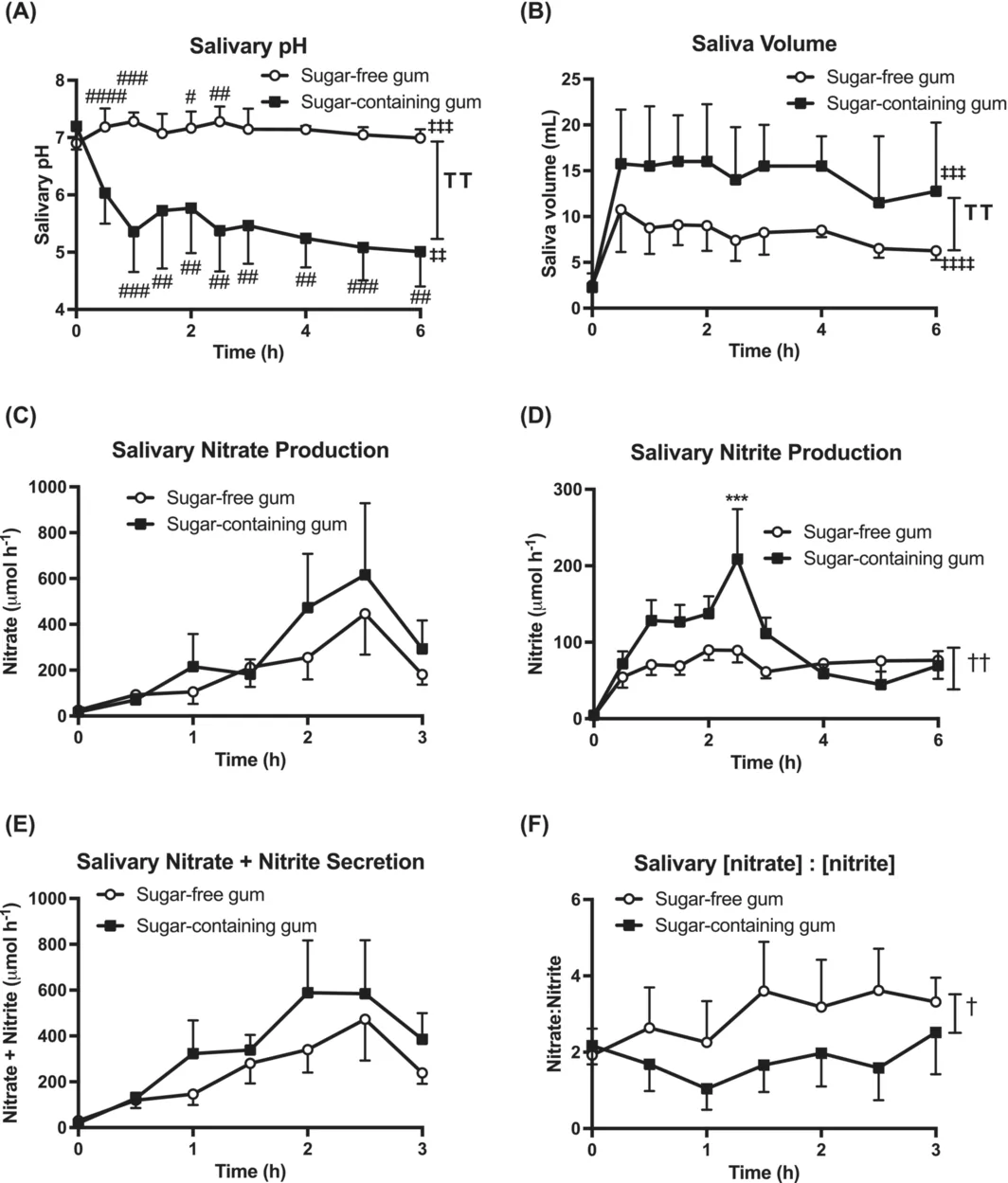
The effect of sugar‐free *vs.* sugar‐containing gum on salivary parameters: (A) salivary pH, (B) saliva volume, (C) salivary nitrate production, (D) salivary nitrite production, (E) salivary nitrate + nitrite production and (F) salivary nitrate:nitrite ratio. Data are expressed as median ± IQR (A,B), and as mean ± SEM (C–F), *n* = 14 for (A, B, D: 6 h), *n* = 8 for (C, E, F: 3 h). Significance shown as ^T T^ *p* < .01, Wilcoxon matched pairs signed rank test, followed by ^‡‡^ *p* < .01, ^‡‡‡^ *p* < .001, ^‡‡‡‡^ *p* < .0001 for within‐group comparison to baseline overall, and ^#^ *p* < .05, ^##^ *p* < .01, ^###^ *p* < .001, ^####^ *p* < .0001 for individual time points, or ^††^ *p* < .01, ^††††^ *p* < .0001 as analysed by two‐way ANOVA, followed by ***p < .001, for Sidak's post‐test for individual time points.

On inspection of the data for the second cohort, which extended out to 6 h, we found that the salivary [nitrate] was many times greater than that of the first cohort (3 h). For sugar‐free gum, the salivary [nitrate] was ~7‐fold greater for the second *vs*. the first cohort, (mean ± SEM: 10524 ± 1321 μM *vs*. 1557 ± 367 μM, respectively; difference between the means: 8967 μM [95% CIs 4579 to 13354], *p* = .0004, and similar for median ± IQR: 10821 ± 5386 *vs*. 1527 ± 1250 μM, respectively). For sugar‐containing gum, the salivary [nitrate] was ~4–5–fold greater for the secondnd (3 h) *vs*. the first (6h) cohort, (mean ± SEM: 5973 ± 613 μM *vs*. 1563 ± 408 μM, respectively; difference between the means: 4410 μM [95% CIs 2185 to 6634], *p* = .0002, and similar for median ± IQR: 6248 ± 1742 *vs*. 1272 ± 1638 μM, respectively).

We reanalysed a sample of the second cohort samples for salivary [nitrate] but obtained similar elevated results. This suggested nitrate contamination of the saliva samples in the second cohort, possibly related to a change in the collection tubes, and we have therefore not included those second cohort results for parameters relating to salivary nitrate (salivary nitrate amount, salivary nitrate + nitrite amount and salivary nitrate:nitrite ratio), just presenting the data for the first cohort for these, which only extend out to 3 h. Salivary [nitrite] was similar between the first (3‐h) and second (6‐h) cohorts, and all the data were included in the analysis relating to salivary nitrite production.

Salivary nitrate production was not different between the conditions (*p* = .2); see Figure 3C. However, salivary nitrite production was increased by sugar‐containing *vs*. sugar‐free gum (*p* < .01) with a mean difference of 30.0 ± 11.0 μmol h^−1^ (from 66.3 ± 7.8 to 96.2 ± 18.4 μmol h^−1^); see Figure 3D. There was no significant increase in salivary nitrate + nitrite secretion (*p* = .1); see Figure 3E. The salivary nitrate:nitrite ratio was decreased by sugar‐containing gum (*p* < .05) with a mean difference of 1.1 ± 0.5; see Figure 3F.

### 3.2 Plasma

There was no significant difference in plasma [nitrate] between sugar‐containing *vs*. sugar‐free gum (*p* = .2); see Figure 4A. However, plasma [nitrite] was increased from 900 ± 321 to 1122 ± 428 nmol L^−1^ by sugar‐containing *vs*. sugar‐free gum (*p* = .0001), with the peak difference at *t* = 2.5 h: +406 nmol L^−1^ (95% CI 7.3 to 805) *p* = .001; see Figure 4B.

**FIGURE 4:**
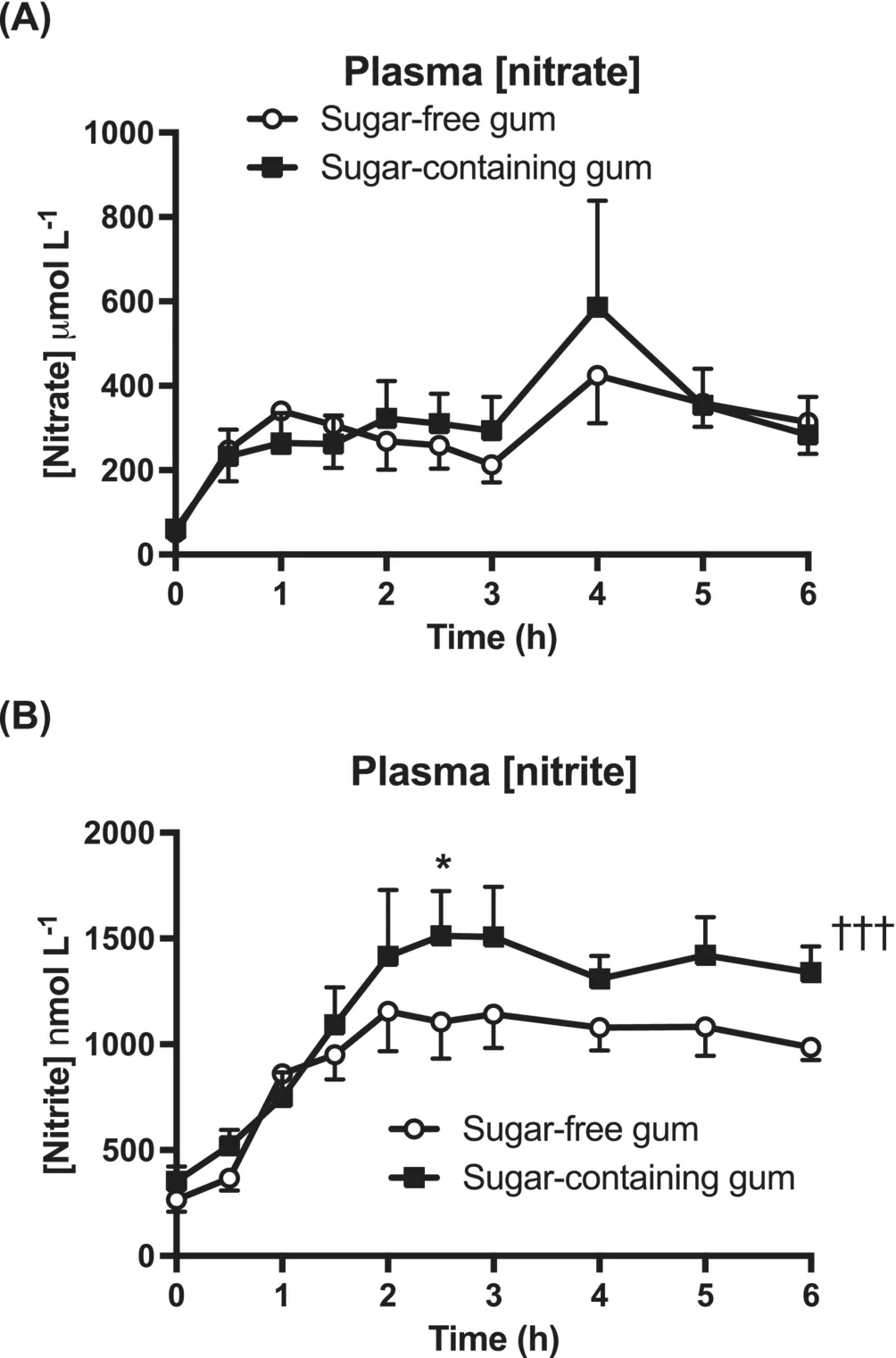
The effect of sugar‐free *vs.* sugar‐containing gum on (A) plasma [nitrate] and (B) plasma [nitrite]. Data are expressed as mean ± SEM, *n* = 14. Significance shown as ^†††^ *p* < .001 as analysed by two‐way ANOVA, followed by *p < 0.05 for Sidak's post‐test.

### 3.3 BP

Systolic BP (SBP) and diastolic BP (DBP) were significantly decreased with sugar‐containing *vs*. sugar‐free gum by 2.7 ± 0.5 and 1.9 ± 0.5 mmHg, respectively (both *p* < .0001); see Figure 5A,B, respectively.

**FIGURE 5:**
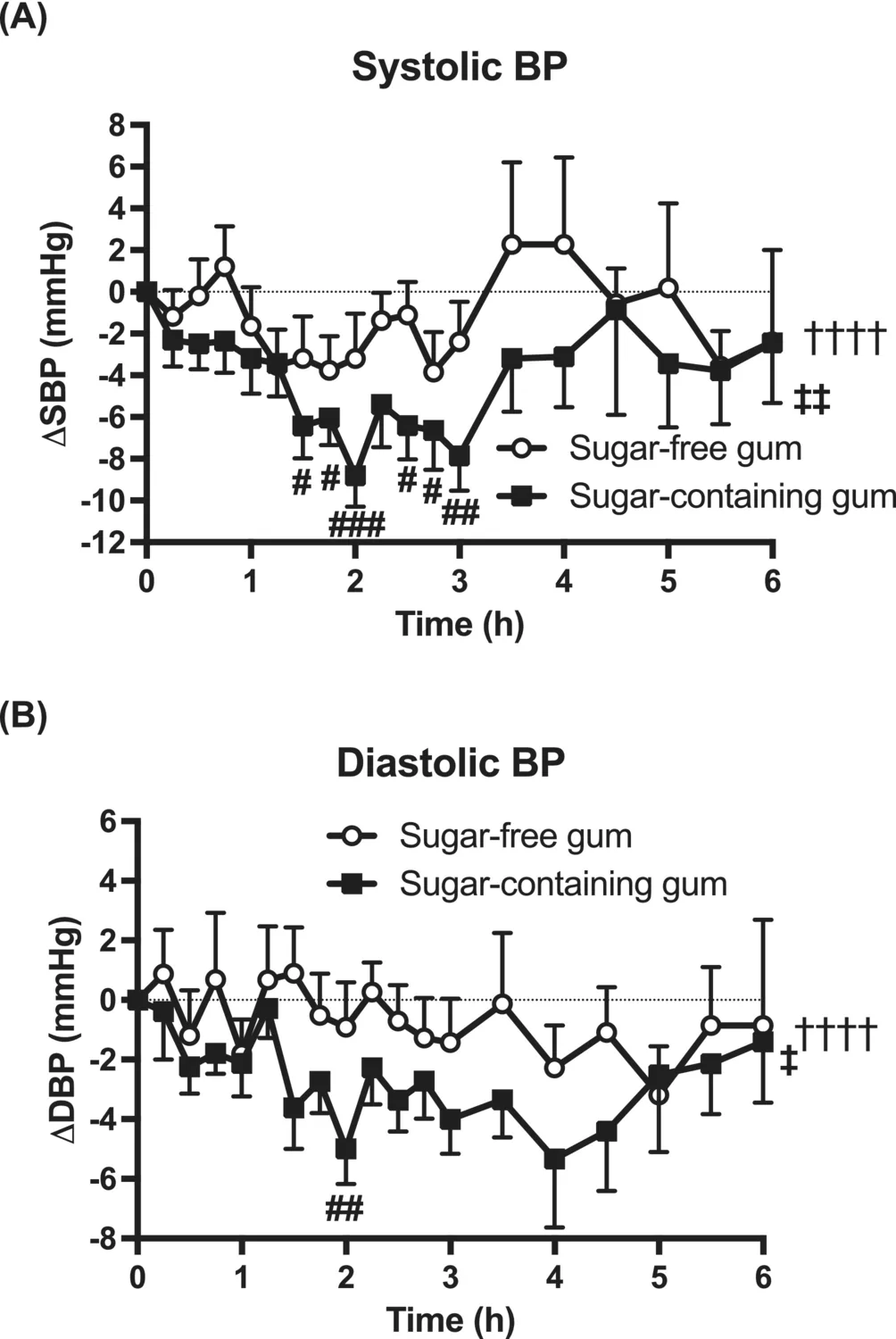
The effect of sugar‐free and sugar‐containing gum on changes from baseline in blood pressure (BP), (A) systolic BP (SBP) and (B) diastolic BP (DBP). Data are expressed as mean ± SEM, *n* = 14. Significance shown as ^††††^ *p* < .0001 as analysed by two‐way analysis of variance (ANOVA), followed by ^‡^ *p* < .05, ^‡‡^ *p* < .01, for within‐group comparison to baseline overall (one‐way ANOVA), and ^#^ *p* < .05, ^##^ *p* < .01, ^###^ *p* < .001 for individual time points *vs.* baseline for post‐tests for individual time points.

## 4 DISCUSSION

In this study we found that chewing sugar‐containing gum compared to sugar‐free gum was effective in significantly lowering the salivary pH throughout the duration of the study. Decreased salivary pH was associated with a 45% elevation in salivary nitrite production (Figure 3D), a 25% increase in plasma [nitrite] (Figure 4B) and enhanced BP lowering by dietary nitrate (Figure 5). A likely mechanism is that the lower salivary pH induced by sugar‐containing gum enhances the nitrate reductase activity of the commensal bacteria, as reflected by the significantly increased salivary nitrite production from *t* = 1 h. The increased saliva volume did not alter the salivary nitrate amount, the combined salivary nitrate + nitrite amount, or the plasma [nitrate]. Instead, the salivary nitrate:nitrite was decreased, reflecting the increased salivary nitrite production in the absence of a significant increase in salivary nitrate. The integrated series of pathways and mechanisms are summarized in Figure 6.

**FIGURE 6:**
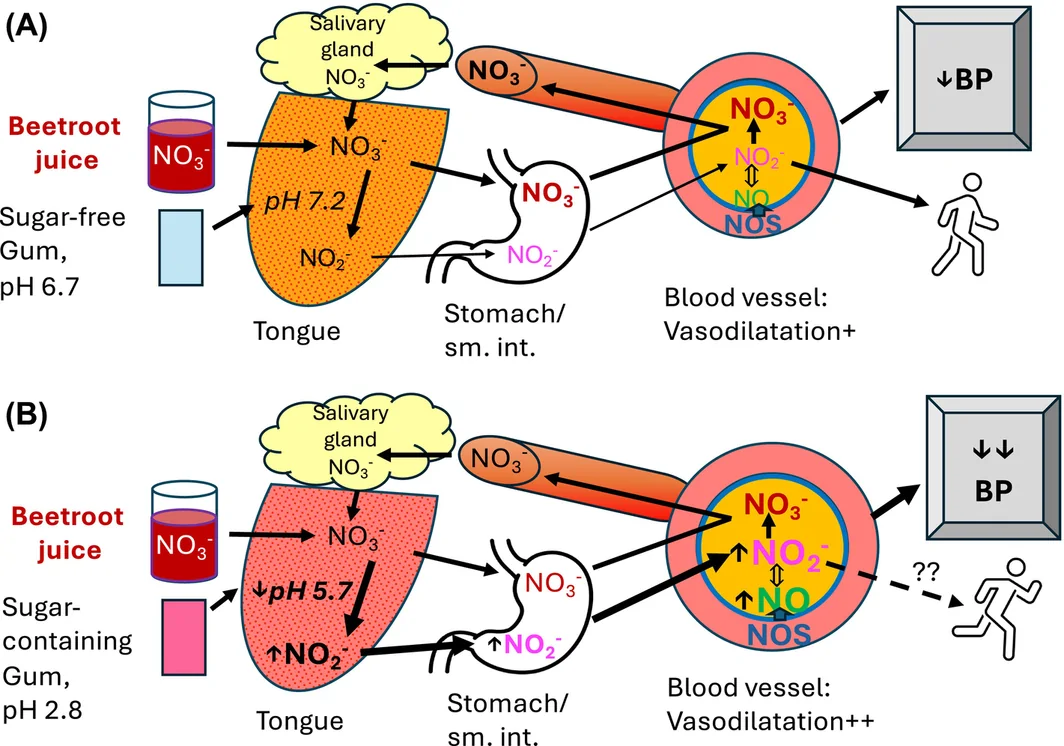
The acute effect of (A) sugar‐free gum *vs.* (B) sugar‐containing acid gum on lowering salivary pH, increasing nitrate (NO_3_^−^) and reduction to nitrite (NO_2_^−^) by bacterial nitrate reductases on the tongue, elevating circulating plasma [nitrite], which is an alternative source of nitric oxide (NO) in the blood vessel (besides nitric oxide synthase (NOS)) resulting in further vasodilatation and blood pressure (BP) lowering. In addition, there is a proposed potential effect on exercise performance. Salivary nitrate is derived from dietary nitrate in beetroot juice (exogenous) and the enterosalivary circulation of nitrate, whereby nitrate is absorbed from the small intestine into the circulation, alongside NOS‐derived nitrite and nitrate (endogenous) and is delivered to and secreted by the salivary glands.

Consistent with our findings, Ong et al. recently tested chewing gum after a nitrate‐rich meal (nitrate [180 mg], from spinach [200 g]) in 20 healthy volunteers.^30^ They found significant decreases in salivary [nitrate] and [nitrite], and a trend to a decrease in the ‘nitrate reductase ratio’, which they defined as *‘The ability of each participant's oral microbiome to reduce salivary nitrate to nitrite … calculated as … (100 x [nitrite]/([nitrate] + [nitrite]))’*, accompanied by significant *increases* in systolic and diastolic BP with chewing compared to no chewing. They used a commercially available fruit‐flavoured, sugar‐free chewing gum, probably like the sugar‐free gum we used. Thus, whilst they did not test the effect of their gum on salivary pH, it would be anticipated to have a similar effect on increasing salivary pH as we found with our sugar‐free gum, compared to baseline (*p* = .001 overall, see Figure 3A), from pH 6.90 (6.71 to 7.13), median (IQR), to pH 7.28 (7.13 to 7.44) and pH 7.28 (6.98 to 7.54) at 1 and 2.5 h, respectively. This may have accounted for the lack of BP‐lowering from baseline that we observed with the sugar‐free gum. We suggest that such a potential increase in salivary pH with the gum used by Ong et al. might account for the trend to a decreased nitrate reductase ratio and increased BP.

We previously found that addition of grapefruit juice to beetroot juice significantly increased salivary pH, decreased salivary nitrite production, increased salivary nitrate:nitrite ratio and decreased plasma [nitrite].^12^ Our grapefruit juice study,^12^ the study of Ong et al.^30^ and our current study represent acute whole‐body/systemic studies. By contrast, studies employing isolated models, using oral installations or incubations of pH‐modifying solutions appear to generate the opposite relationship between oral pH and nitrate reduction. For example, Bojic DV et al. (2004) found that nitrate reduction increased linearly with increasing pH 5.5–8.0, with 2‐min oral sodium nitrate incubations (10 mg‐N dm^−3^) in six healthy volunteers.^19^ However, only ~6% of the nitrate was reduced to nitrite. We previously demonstrated that salivary [nitrite] constitutes ~33% of total salivary [nitrate] and [nitrite],^9^ and, similarly, in the current study, the overall average salivary nitrate:nitrite ratios were 2.9 ± 0.7 and 1.8 ± 0.5 with sugar‐free and sugar‐containing gum, respectively. Also, some of the participants in the study by Bojic et al. were smokers.^19^ Bailey et al. demonstrated that cigarette smokers have blunted salivary nitrate reduction to nitrite, possibly through inhibition of salivary nitrate uptake by elevated circulating thiocyanate.^31^ Such diminished nitrate reduction to nitrite in smokers may remove a protective mechanism against gastroduodenal ulcers,^32^, ^33^ and via decreased plasma nitrite, increase platelet aggregation/cardiovascular risk.

van Maanen JM et al. found that salivary nitrite concentration at 0, 30, 60 and 120 min after nitrate ingestion (3.8 mmol) was significantly higher at 30 min only (*p* = .03; Mann–Whitney *U* test), with volunteers (*n* = 5) chewing pH‐regulating gum on Day 2 (generating a salivary pH ~7.3) *vs*. chewing control gum on Day 1 (generating a salivary pH ~6.8) but was numerically lower at all other timepoints.^20^ Again, a lower proportion (~16%) of salivary nitrate was reduced to nitrite. More recently, Cocksedge et al. increased oral pH in healthy volunteers by rinsing for 2 min every 7.5 min with 30‐mL 4.2‐M NaHCO3 solution (pH 8.1 ± 0.1) and found significant increases in salivary and plasma [nitrite] *vs*. normal pH solution (pH 7.3 ± 0.1), with an associated decrease in salivary [nitrate] assessed at 1, 2 and 3 h.^21^

In support of our findings, Vavilova and lu Petrovich found maximal nitrate reductase activity at a lower pH: pH 6.5–7.0 using whole saliva ex vivo.^34^ While Hohensinn et al. found that chronic ingestion of beetroot juice by 46 volunteers over 15 days was found to increase salivary pH from 7.0 to 7.5, this was associated with a general increase in both salivary [nitrate] and [nitrite].^13^ As noted above, changes in the oral microbiota are induced by chronic nitrate ingestion,^14^ which likely contribute to increased salivary pH,^15^ conferring beneficial effects on periodontal/gingival health.^13^, ^17^, ^18^ While Burleigh et al. also found a similar increase in salivary pH (from 7.13 to 7.39) associated with changes in the abundance of some of the lingual bacteria following 7 days' nitrate supplementation, there were no differences in salivary or plasma nitrite or BP responses.^16^ Therefore, the design of the study, that is, acute *vs*. chronic and isolated oral installation/incubation model *vs*. fully systemic experiments appear to produce different results. Either way, it appears to be possible to manipulate the nitrate–nitrite–NO pathway to bring about an increased physiological effect from a dietary nitrate load.

Our findings are directly translatable for individuals who wish to acutely enhance nitrate bioactivation and associated physiological effects following dietary nitrate (e.g., beetroot juice) ingestion. Besides BP lowering this would likely include enhancing exercise performance, though this remains to be tested specifically. However, there is a reasonably large body of evidence in support of dietary nitrate's effect per se in enhancing exercise performance.^35^ Note that the elevated nitrate reduction with decreased salivary pH may only be a transient/acute effect: The BP‐lowering effect wore off in the sixth hour‐post ingestion/of chewing, and chronic modulation of salivary pH may have the opposite effect, for example, with changes in the oral microbiota, as described above. Also, using a sugar‐containing chewing gum has limitations in patients, for example, with cardiovascular disease, who have a high prevalence of co‐existing diabetes mellitus. Given these issues, we are not explicitly recommending sugar‐containing gum as a general approach to enhance the physiological effects of the nitrate–nitrite–NO pathway, for example, on BP lowering; we employed this method to explore and better understand the mechanistic associations between salivary pH and salivary nitrate reduction. However, as an observation, the classic culinary tradition of having a sweet dessert (which may be in the form of fruit) towards the end of a meal, after a starter and a main course that often contain nitrate‐rich salad leaves and vegetables, may enhance salivary nitrate reduction to nitrite, by lowering salivary pH, and enhance the physiological effects such as BP lowering. Nonetheless, we caution against an excess of sugar, resulting in a substantial systemic elevation in glucose/hyperglycaemia, which we and others have found may blunt the BP‐lowering effect of dietary nitrate, also apparent in patients with type 2 diabetes mellitus.^23^, ^36^, ^37^ However, Burleigh et al. proposed that nitrate may represent a nutritional strategy to diminish the risk of developing acidity‐related oral health conditions: They found that consumption of nitrate‐containing beetroot juice (pH 4.0) attenuated the acidification of salivary pH following a sugar (30 g) containing drink (pH 3.2), albeit in the context of 90‐min submaximal running with exercise‐induced dehydration.^38^ Similarly, Rosier et al. found that salivary pH dropped to a lesser degree (by pH 0.23) with a sucrose rinse following nitrate‐rich beetroot juice *vs*. placebo.^39^

### 4.1 Limitations

The study involved healthy volunteers. Therefore, the results should be considered within the context of physiological mechanisms; any extrapolation to the potential effects in patients with hypertension is hypothesis generating.

As presented in the methods section, we recruited an initial eight volunteers before deciding on a final sample size of 14. Furthermore, for the final six volunteers, we elected to collect data until *t* = 6 h, based on the initial findings. Although we consider an iterative approach to be valid for an exploratory, mechanistic, experimental medicine study such as this, it does carry limitations. For example, the final sample size was not defined a priori but was decided based on data from the first eight volunteers. As stated by Jones et al, this approach should be used with caution as in theory it can lead a researcher to discontinue a study at the point of interim analysis if the results are statistically significant at the point of interim analysis (which may have occurred due to chance).^40^ Although we had adequately powered the initial part of the study to detect a difference in salivary nitrite production, which was achieved.

We have not presented data for salivary nitrate amount or concentration for volunteers 9–14 as the results were extremely high (values up to 44,000 μmol L^−1^), likely to be due to contamination.

In this study, to provide a constant pH‐altering mechanism, we used chewing gum, which has multiple ingredients, for example, acesulfame‐K and aspartame. Aspartame has been shown to decrease systolic BP in rats^41^; however, we found no data supporting a similar effect in humans (a prospective randomized study protocolised BP measurement, but did not report the data within the manuscript or supplemental data).^42^ However, both chewing gums used in this study (Hubba Bubba®, Wrigley's Extra®) contain both acesulfame‐K and aspartame, so any BP effect should be controlled for. Whilst the acute effect of altering salivary pH on nitrate reduction likely represents a direct effect on nitrate reductase activity, an acute alteration of the oral microbiome or bacterial activity is possible but was not tested here.

In conclusion, to our knowledge, we have provided the first evidence, in a systemic human study employing the enterosalivary circulation, that specifically and acutely lowering salivary pH, here with a sugar‐containing gum, augments salivary nitrate reduction to nitrite, supported by evidence of the consequent rise in plasma [nitrite], and an enhancement of the BP‐lowering effect of dietary nitrate. This complements our previous study, whereby an intervention (grapefruit juice), which was incidentally found to elevate salivary pH, had the opposite effect: lowering salivary nitrate reduction to nitrite and (lowering) plasma [nitrite].^12^ This appears to contrast with studies employing isolated models, i.e., using oral installations or incubations of pH‐modifying solutions, which generate the opposite relationship between salivary pH and nitrate reduction, and, by their nature, are also not supported by data on circulating plasma [nitrite]. This renewed understanding of the relationship between salivary pH and the nitrate‐nitrite‐NO pathway is key to optimizing its beneficial physiological effects.

## AUTHOR CONTRIBUTIONS

Andrew J. Webb conceived and designed the study. Andrew J. Webb, Harriet Clift, Callum Hill, Nur Mousa, Navanithan Arun Jayaraj, Jasmine Quraishi, Charlotte E. Mills and Kevin O'Gallagher performed data collection and data analysis. Andrew J. Webb, Charlotte E. Mills and Kevin O'Gallagher were involved in data interpretation and drafting (and revision) of the manuscript. All authors have given final approval of this manuscript.

## CONFLICT OF INTEREST STATEMENT

A.J.W. holds shares in HeartBeet Ltd, which receives a royalty from James White Drinks Ltd., which manufactures the beetroot juice used in this study. The other authors have no competing interests to declare.

## Data Availability

The data that support the findings of this study are available from the corresponding author upon reasonable request.
